# A concatenated LDPC-marker code for channels with correlated insertion and deletion errors in bit-patterned media recording system

**DOI:** 10.1371/journal.pone.0270247

**Published:** 2022-07-08

**Authors:** Tianbo Xue

**Affiliations:** Department of Electronic and Information Engineering, The Hong Kong Polytechnic University, Hung Hom, Hong Kong; National University of Sciences and Technology, PAKISTAN

## Abstract

Most synchronization error correction codes deal with random independent insertion and deletion errors without correlation. In this paper, we propose a probabilistic channel model with correlated insertion and deletion (CID) errors to capture the data dependence applicable to the bit-patterned media recording (BPMR) system. We also investigate the error performance and decoding complexity of a concatenated LDPC-marker code over the CID channel. Furthermore, we modify the forward backward decoding algorithm to make it suitable for the CID channel, and elaborate it based on a two-dimensional state transition diagram. Compared with the conventional marker coding scheme dealing with random errors, the concatenated LDPC-marker code takes into account the dependence between synchronization errors, improves the error performance, and reduces the decoding complexity. The BER performance of the concatenated LDPC-marker code is improved by more than 50% on average, and the decoding time is reduced by nearly 35% when the LDPC code (*n* = 4521, *k* = 3552) and the marker code (*N*_*m*_ = 2, *N*_*c*_ = 30) are used over the CID channel.

## I. Introduction

Channels impaired by insertion, deletion and substitution errors were first considered in 1961 [1]. Insertions and deletions are categorized as synchronization errors because they affect the synchronization of a communication channel. Recently, channels with synchronization errors have attracted renewed interest because of applications such as DNA-based data storage [2], image watermarking [3], and video digital watermarking [4]. Conventional error correction codes such as low-density parity-check (LDPC) codes [5] and Reed-Solomon codes [6], which are designed to combat noise and substitutions, cannot correct insertions and/or deletions.

Many coding schemes have been proposed for channels impaired by random insertion, deletion and substitution errors in the past few decades [7]. Synchronization error correction mechanisms have shown promising results by adopting a concatenated scheme. An LDPC code with inserted markers can correct multiple insertions and deletions in [8]. RS codes are concatenated with marker codes in [9] to correct residual errors in the output from the marker decoder. The authors in [10] constructed a family of synchronization error correction codes through concatenation, and provided efficient encoding and decoding algorithms. A concatenated LDPC-trellis code with a high information rate is constructed in [11]. In [12], the authors proposed a coding scheme consisting of a 2-D marker code and an irregular LDPC code to achieve excellent decoding performance under a non-iterative scenario against insertions and deletions in racetrack memories.

However, the concatenated coding schemes mentioned above are developed for channels with random independent errors. Very few papers focus on codes correcting correlated errors. In fact, correlated insertion and deletion errors exist in some practical applications, such as BPMR systems. In BPMR, deletions and insertions often occur in pairs (i.e., a deletion is followed by an insertion in a latter position, and vice-versa) [13]. In particular, experiments are conducted to provide written-in error statistics and experimental data is provided, where synchronization is first lost due to an insertion and then resynchronize following a deletion, and vice-versa. It is further noted that the subsequent synchronization error is more likely to occur at the position closer to the preceding one and the error probability decreases with the distance. From these circumstances, insertions and deletions are correlated rather than independent and random in the writing process of BPMR.

Several channel models suitable for the writing process of BPMR have been proposed. Dependence of written-in errors was considered by Mazumdar et al. in [14], but the only case studied was a bit erroneously changed to the preceding value when binary data was written on the medium. The authors in [15] proposed a write channel model with input-dependent noise for BPMR, and theoretically analyzed the lower and upper bounds of the information rate. A recent paper [16] presented a channel model with correlated insertion and deletion errors adapted to BPMR systems. The channel model is fully described by a finite-state machine, i.e., the current event (insertion, deletion or transmission) is completely dependent on the previous event. Although the occurrence of consecutive insertion/deletion errors is prohibited, it does not guarantee the insertion event and the deletion event to appear in pairs. Moreover, the insertion and deletion error probability after a synchronization error is fixed to 0.5, which does not fit the fact that the subsequent synchronization error is more likely to occur at a closer position of the preceding one in BPMR.

In BPMR writing process, 1) a deletion is followed by an insertion in a latter position, and vice-versa; 2) the subsequent synchronization error probability is more likely to occur at the position closer to the preceding one and decreases with the distance. As far as we know, no channel models have been proposed for BPMR systems that considers both of the above error statistics. For the above reasons, we propose a more accurate channel model that takes into account all error statistics in BPMR writing process.

In this paper, we first propose a probabilistic channel model with correlated insertion and deletion errors, in addition to substitution errors. The channel model captures, for example, the data dependence adapted to the write channel in BPMR more accurately. Then, we modify the forward-backward algorithm to make it suitable for the proposed channel. Finally, we compare the error performance of the concatenated LDPC-marker coding scheme with the conventional marker coding scheme without considering the dependency between synchronization errors over the proposed channel. The paper is organized as follows. Sect. II introduces the proposed channel model, the concatenated LDPC-marker code, and the encoding and decoding steps. Sect. III describes the details of the modified forward-backward algorithm based on a two-dimensional transition diagram. Sect. IV presents simulation results and discussion. Concluding remarks and future work are given in Sect. V.

## II. Concatenated LDPC-marker code

This section starts with a binary channel with random errors and the proposed probabilistic channel model with correlated insertion and deletion errors, followed by a description of the encoding and decoding of the concatenated LDPC-marker code.

### BSID channel with random errors

**Fig 1:**
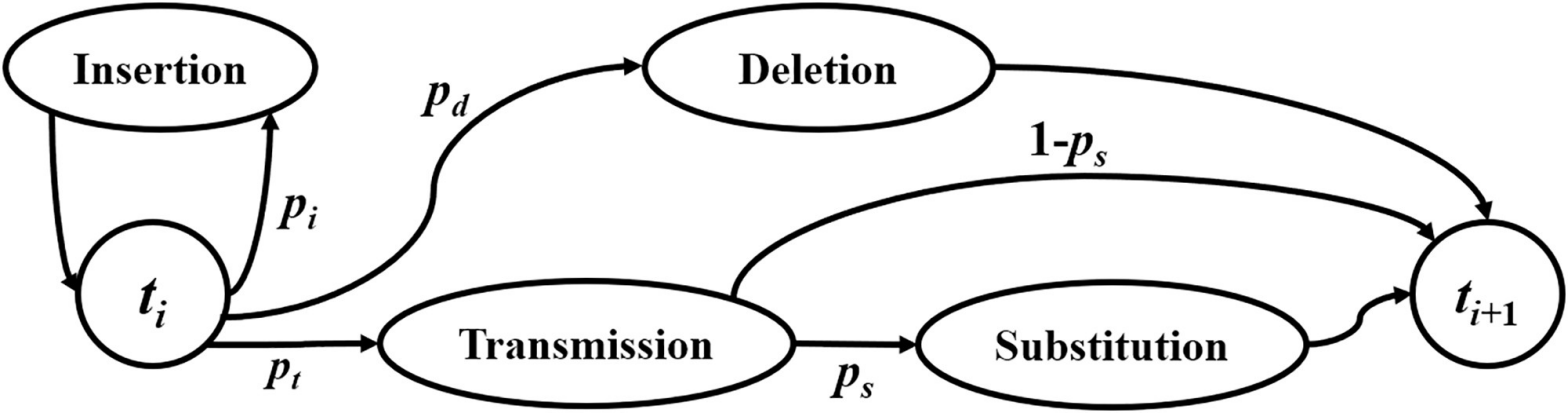
BSID channel model.

A channel with random independent insertion, deletion and substitution errors proposed by Davey and MacKay [17] is depicted in Fig 1. For the binary case, this channel is referred to as binary substitution/insertion/deletion (BSID) channel. It can be regarded as a binary symmetric channel (BSC) with synchronization errors. For each transmitted bit *x*_*k*_, three possible events may occur: (1) *x*_*k*_ is deleted with probability *p*_*d*_; (2) one more bit is transmitted before *x*_*k*_ with probability *p*_*i*_; (3) *x*_*k*_ is transmitted with probability *p*_*t*_ = 1 − *p*_*d*_ − *p*_*i*_. Moreover, the transmitted bit may be substituted with probability *p*_*s*_. The operation repeats until all bits are sent. Insertions and deletions are random without correlation in this channel.

### Proposed CID channel with correlated errors

Here, we propose a probabilistic channel with correlated insertion and deletion (CID) errors, i.e., an insertion and a deletion occur in pairs while substitution errors occur randomly within a sequence. Such a channel is referred to as a correlated insertion/deletion (CID) channel.

Given a transmitted sequence $x_{1}^{t}=\left(x_{1},x_{2},\ldots ,x_{t}\right)\in {\left\{0,1\right\}}^{t}$, we assume synchronization errors occur to $x_{a_{1}},x_{b_{1}},x_{a_{2}},x_{b_{2}},x_{a_{3}},x_{b_{3}},\ldots$ where *a*_1_ < *b*_1_ < *a*_2_ < *b*_2_ < *a*_3_ < *b*_3_ < ⋯. We also call $\left(x_{a_{n}},x_{b_{n}}\right)$ (*n* = 1, 2, …) a synchronization error bit pair.

The characteristics of the proposed probabilistic channel model are as follows.

1. The synchronization error probability of $x_{a_{n}}$ is determined by the blackBSID channel.
2. We assume $x_{a_{n}}$ and $x_{b_{n}}$ suffer from different synchronization errors. In other words, if $x_{a_{n}}$ suffers from an insertion error, $x_{b_{n}}$ will suffer from a deletion error; and vice versa.
3. The synchronization error probability of $x_{b_{n}}$ is determined by the synchronization error type of $x_{a_{n}}$ and the bit separation *l* between $x_{a_{n}}$ and $x_{b_{n}}$. The synchronization error probability of $x_{b_{n}}$ decreases as the separation *l* increases.

We consider the synchronization error bit pair $\left(x_{a_{n}},x_{b_{n}}\right)$. After a synchronization error has occurred to $x_{a_{n}}$, we denote the probability of the synchronization error occurring at $x_{a_{n}+l}$ by $\text{Pr}(x_{b_{n}}=x_{a_{n}+l})$. Since $\text{Pr}(x_{b_{n}}=x_{a_{n}+l})$ is a decreasing function of *l*, we assume that $\text{Pr}(x_{b_{n}}=x_{a_{n}+l})$ (*l* = 1, 2, …) forms a geometric progression with a common ratio of *r* < 1, i.e.,

$$\begin{array}{c} \text{Pr}\left(x_{b_{n}}=x_{a_{n}+l}\right)=Ar^{l-1};l=1,2,\ldots \end{array}$$ (1)

where *A* is a constant. After a synchronization error has occurred to $x_{a_{n}}$, we use $p_{d}^{\prime }$, $p_{i}^{\prime }$ and $p_{t}^{\prime }$ to represent the new deletion probability, new insertion probability and new transmission probability, respectively, for *x*_*a*_*n*_+ *l*_. Moreover, they are given by Eqs (2) to (4). The probabilities will be reset to *p*_*d*_, *p*_*i*_ and *p*_*t*_ once a synchronization error occurs to $x_{b_{n}}$. We further assume that random substitution errors always exist with an error probability *p*_*s*_. The above operation repeats until all transmitted bits are sent.

$$\begin{array}{ccc} p_{d}^{\prime } & = & \left\{ \begin{array}{cc} Ar^{l-1} & \text{if}\;\text{an}\;\text{insertion}\;\text{error}\;\text{has}\;\text{occurred}\;\text{to}\;x_{a_{n}}\;\\ 0 & \text{if}\;\text{a}\;\text{deletion}\;\text{error}\;\text{has}\;\text{occurred}\;\text{to}\;x_{a_{n}} \end{array}\right. \end{array}$$ (2)

$$\begin{array}{ccc} p_{i}^{\prime } & = & \left\{ \begin{array}{cc} 0 & \text{if}\;\text{an}\;\text{insertion}\;\text{error}\;\text{has}\;\text{occurred}\;\text{to}\;x_{a_{n}}\;\\ Ar^{l-1} & \text{if}\;\text{a}\;\text{deletion}\;\text{error}\;\text{has}\;\text{occurred}\;\text{to}\;x_{a_{n}} \end{array}\right. \end{array}$$ (3)

$$\begin{array}{ccc} p_{t}^{\prime } & = & 1-p_{i}^{\prime }-p_{d}^{\prime }=1-Ar^{l-1}. \end{array}$$ (4)

### Encoder

**Fig 2:**
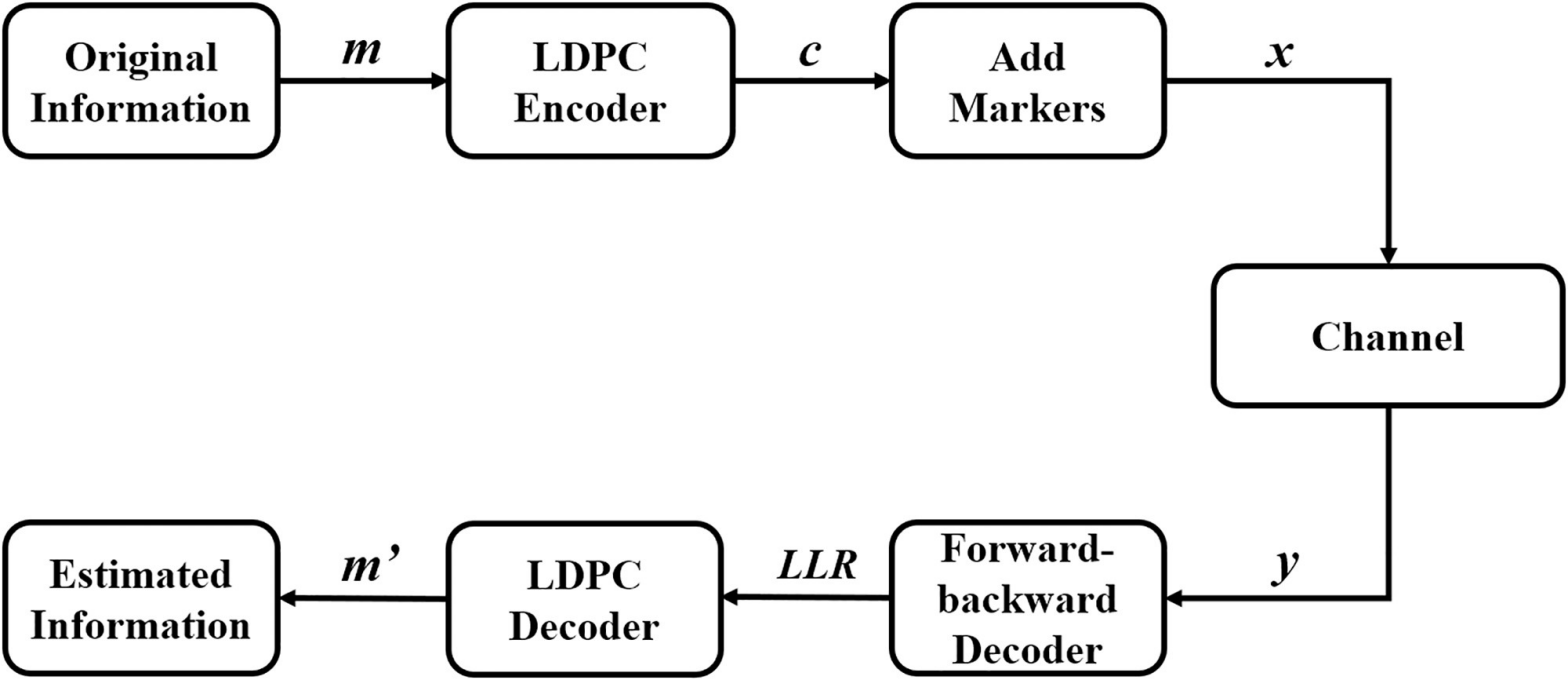
Flow chart of the encoding and decoding of the concatenated LDPC-marker code.

The presented scheme consists of an outer LDPC code and an inner marker code, as shown in Fig 2. The outer code is used to correct errors while the role of the inner marker code is to maintain synchronization. Encoding is implemented in two steps: the message is first encoded into an outer LDPC code and then regular markers [18] with length *N*_*m*_ are inserted periodically every *N*_*c*_ LDPC code bits. For an LDPC code with length *N*, the regularly inserted markers divide the LDPC code into *N*/*N*_*c*_ segments (assuming *N*/*N*_*c*_ is an integer). Each of the segment consists of *N*_*c*_ + *N*_*m*_ bits where the first *N*_*m*_ bits are the marker bits and the last *N*_*c*_ bits are the LDPC code bits. The content and position of the periodical markers in the transmitted sequence are known to and used by the receiver to maintain synchronization. The encoded sequence after the two encoding steps is ready to be transmitted through the channel. The code rate of the concatenated code is *R* = *R*_*C*_ ⋅ *R*_*M*_, where *R*_*C*_ and $R_{M}=\frac{N_{c}}{N_{c}+N_{m}}$ are the code rates of the LDPC code and the marker code, respectively.

### Inner marker decoder

We denote $x_{1}^{t}=\left(x_{1},x_{2},\cdots ,x_{t}\right)$, *x*_*i*_ ∈ {0, 1} for *i* = 1, 2, …, *t* and $y_{1}^{r}=\left(y_{1},y_{2},\ldots ,y_{r}\right)$, *y*_*i*_ ∈ {0, 1} for *i* = 1, 2, …, *r* as the transmitted and the received sequences, respectively. At the receiver, the corrupted sequence $y_{1}^{r}$ is first passed into the inner marker decoder. Given the received vector $y_{1}^{r}$, the inner marker decoder makes full use of the information provided by the markers to compute the likelihood $p(y_{1}^{r}|x_{k})$ for *x*_*k*_ ∈ {0, 1} and *k* = 1, 2, …, *t*. To derive the likelihood $p(y_{1}^{r}|x_{k})$, we modify the forward-backward algorithm [19], which will be explained in Sect. IV. The log-likelihood ratio (LLR) value $L(x_{k}|y_{1}^{r})$ is the ratio between the conditional probabilities $\text{Pr}(x_{k}=0|y_{1}^{r})$ and $\text{Pr}(x_{k}=1|y_{1}^{r})$. Let L be the set of positions where the outer LDPC code bits are located in the transmitted sequence. Applying Bayes’ rule, the LLR $L(x_{k}|y_{1}^{r})$ at bit position k∈L can be computed using

$$\begin{array}{c} L\left(x_{k}|y_{1}^{r}\right)=\text{ln}\frac{\text{Pr}(x_{k}=0|y_{1}^{r})}{\text{Pr}(x_{k}=1|y_{1}^{r})}=\text{ln}\frac{p(y_{1}^{r}|x_{k}=0)}{\;\;p(y_{1}^{r}|x_{k}=1)}\;\;k\in L \end{array}$$ (5)

which are used as soft inputs to the outer LDPC decoder for further decoding.

### Outer LDPC decoder

LDPC code is a linear block code with a sparse parity-check matrix *H* in which the number of non-zero entries is small. We apply the sum-product decoding algorithm (also called belief propagation algorithm) to decode the LDPC code by iteratively passing updated LLR messages along the edges between variable nodes and check nodes in the Tanner graph corresponding to *H*. At the end of each iteration, the updated *a posteriori* LLR for each transmitted bit is calculated and an estimated codeword is obtained by making hard decisions based on the LLRs. The LDPC decoder subsequently checks whether the estimated codeword satisfies all check equations. The codeword is successfully decoded if all check equations are satisfied; otherwise, the iteration continues. If the maximum number of iterations is reached and not all check equations are satisfied, the decoding fails.

## III. Modified forward-backward algorithm

In this section, we modify the forward-backward algorithm to make it suitable for the proposed CID channel, taking into account the correlated insertion and deletion errors. We further elaborate a computationally efficient marker decoding algorithm based on a two-dimensional state transition diagram. Finally, the inner marker decoder implements the modified FB algorithm to compute the LLR to initialize the outer LDPC decoder.

### State transition diagram

**Fig 3:**
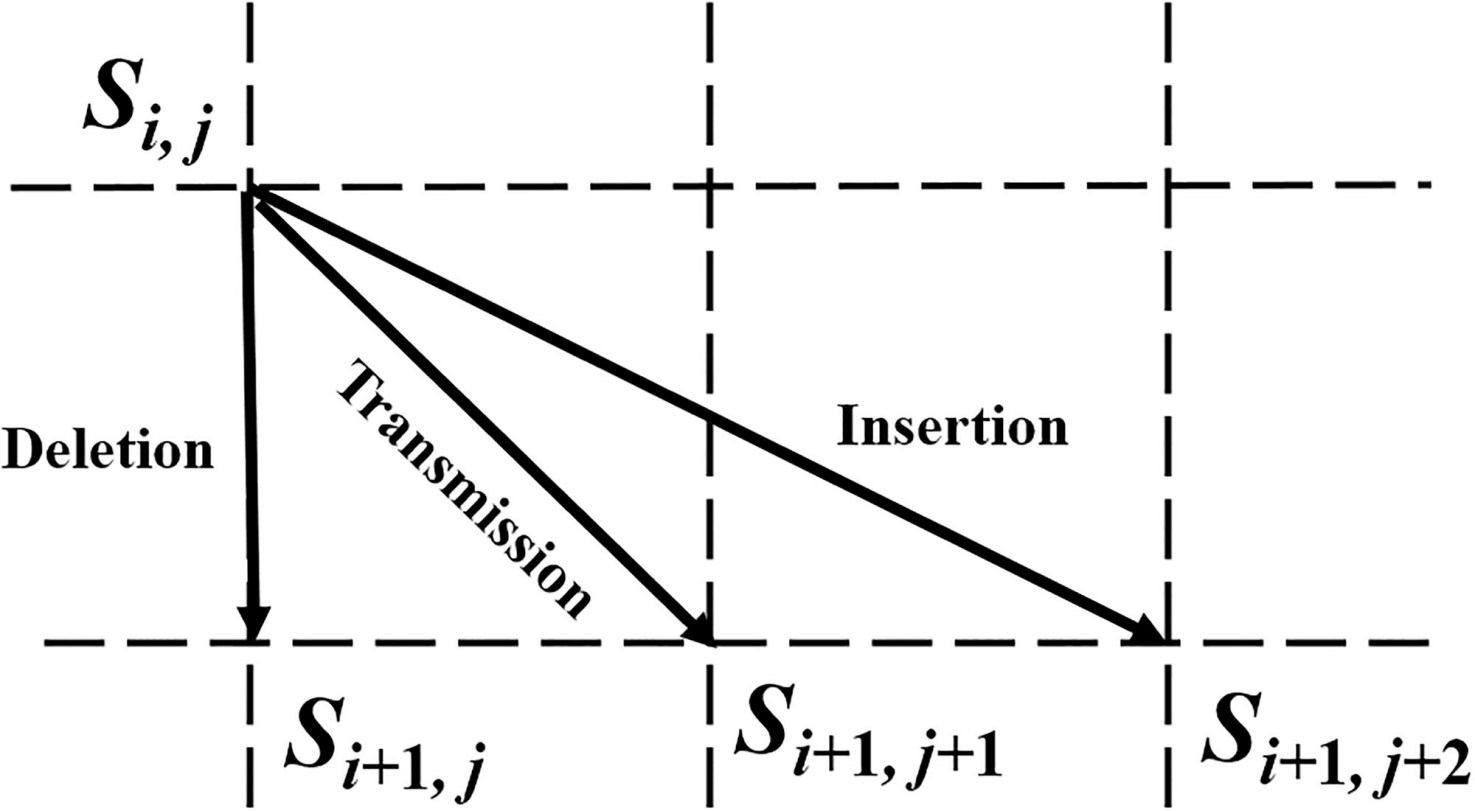
State transition paths for insertion, deletion and transmission when *x*_*i*+ 1_ is transmitted on a two-dimensional grid diagram. *i* increases downwards and *j* increases rightwards.

**Fig 4:**
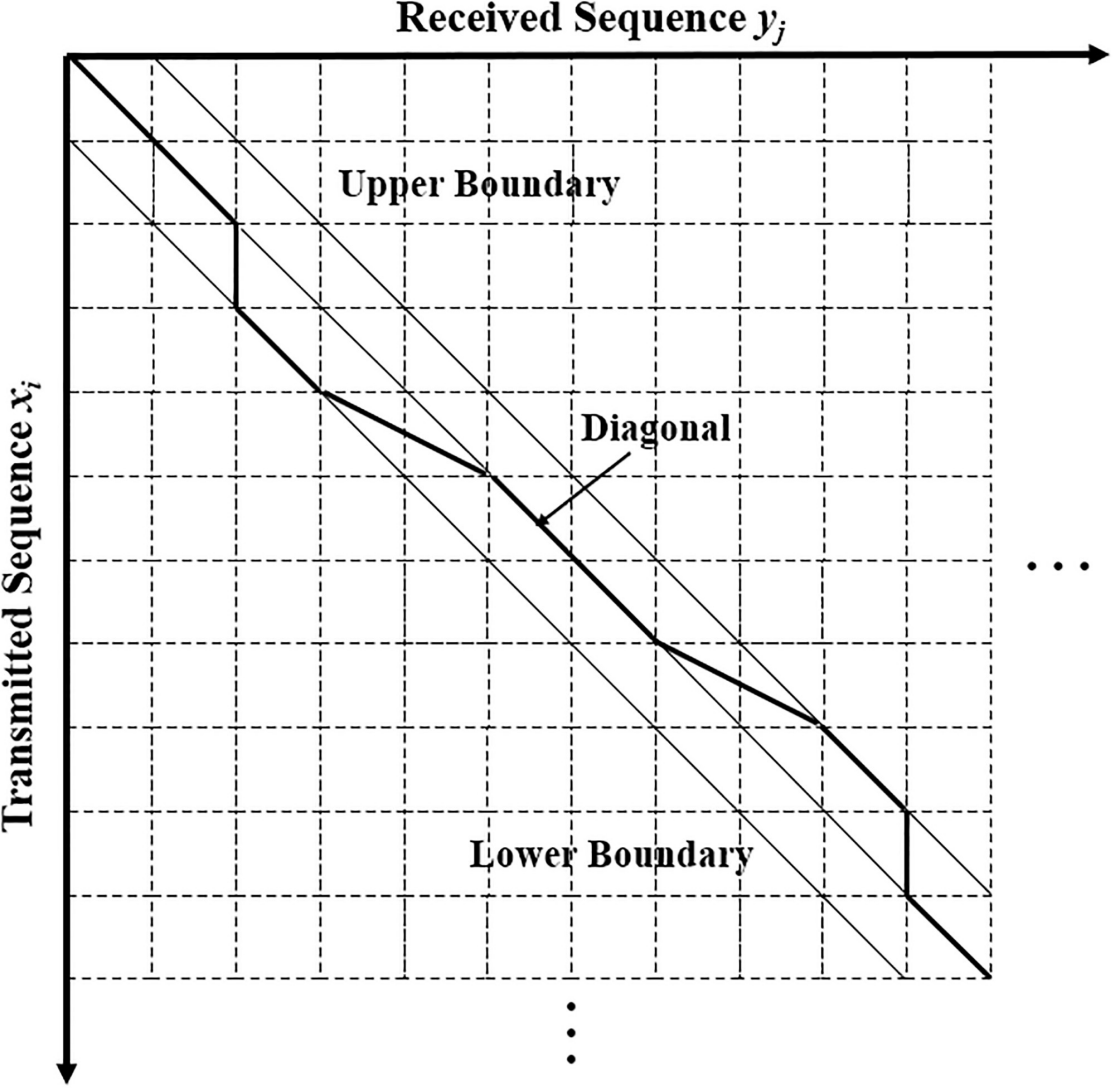
A complete transmission path representing a one-to-one mapping between the received and transmitted sequence on a two-dimensional grid diagram. The actual transmission path represented by the bold line over the CID channel is between the lower and upper boundaries.

We define *S*_0,0_ as the initial state when the transmission begins; and *S*_*t*,*r*_ as the end state when *t* bits have been sent and *r* bits have been received. We also define an intermediate state *S*_*i*,*j*_ when *i* bits (*i* = 1, 2, …, *t*) have been sent and *j* bits (*j* = 0, 1, …, *r*) have been received. State *S*_*i*,*j*_ may transit to *S*_*i*+1,*j*_, *S*_*i*+1,*j*+1_ and *S*_*i*+1,*j*+2_ if there is a deletion error, no deletion/insertion error, and an insertion error, respectively, for the transmitted bit *x*_*i*+1_. The transitions are illustrated in Fig 3. When the state transition paths corresponding to individual transmitted bits are connected together, a complete transmission path like the one shown in Fig 4 is formed. For our CID channel, the complete state transmission path is bounded between the lower boundary (*i* − *j* = 1) and the upper boundary (*j* − *i* = 1).

### Forward algorithm

A transmitted bit *x*_*i*_ is either an LDPC code bit or a marker bit. For an LDPC code bit *x*_*i*_, Pr(*x*_*i*_ = 0) = Pr(*x*_*i*_ = 1) = 0.5 because no information about an LDPC code bit *x*_*i*_ is provided to the decoder and the receiver does not know the value of *x*_*i*_. However, when *x*_*i*_ is a bit from the marker, Pr(*x*_*i*_) is a determined value, i.e., (a) Pr(*x*_*i*_ = 0) = 0 and Pr(*x*_*i*_ = 1) = 1; or (b) Pr(*x*_*i*_ = 0) = 1 and Pr(*x*_*i*_ = 1) = 0. The reason is that the exact value and position of a marker bit *x*_*i*_ are accurately known to the receiver.

We define *T*(*x*_*i*_, *y*_*j*_) as the transmission probability that *x*_*i*_ is transmitted (with/without insertion) and the corresponding received bit is *y*_*j*_, i.e.,

$$\begin{array}{c} T\left(x_{i},y_{j}\right)=\text{Pr}\left(x_{i}\right)\cdot C\left(x_{i},y_{j}\right), \end{array}$$ (6)

where *C*(*x*_*i*_, *y*_*j*_) denotes the probability that the transmitted bit *x*_*i*_ and the corresponding received bit *y*_*j*_ have or do not have the same value. If *x*_*i*_ = *y*_*j*_, *x*_*i*_ has been transmitted without any substitution error and thus *C*(*x*_*i*_, *y*_*j*_) = 1 − *p*_*s*_; otherwise, *x*_*i*_ is substituted and *C*(*x*_*i*_, *y*_*j*_) = *p*_*s*_. Thus, we have

$$\begin{array}{c} C\left(x_{i},y_{j}\right)=\{\begin{array}{cc} 1-p_{s} & \text{if}\;x_{i}=y_{j}\\ p_{s} & \text{if}\;x_{i}\neq y_{j} \end{array}. \end{array}$$ (7)

The forward quantity *α*_*i*,*j*_ is the joint probability that *i* bits are transmitted and the *j* bits $y_{1}^{j}=\left(y_{1},y_{2},\ldots ,y_{j}\right)$ are received. In other words, it is the probability that $y_{1}^{j}$ has been received when the transmission state transits from *S*_0,0_ to *S*_*i*,*j*_. The forward quantity *α*_*i*,*j*_ is given by

$$\begin{array}{c} \alpha_{i,j}=\text{Pr}\left(y_{1}^{j},S_{i,j}\right). \end{array}$$ (8)

**Fig 5:**
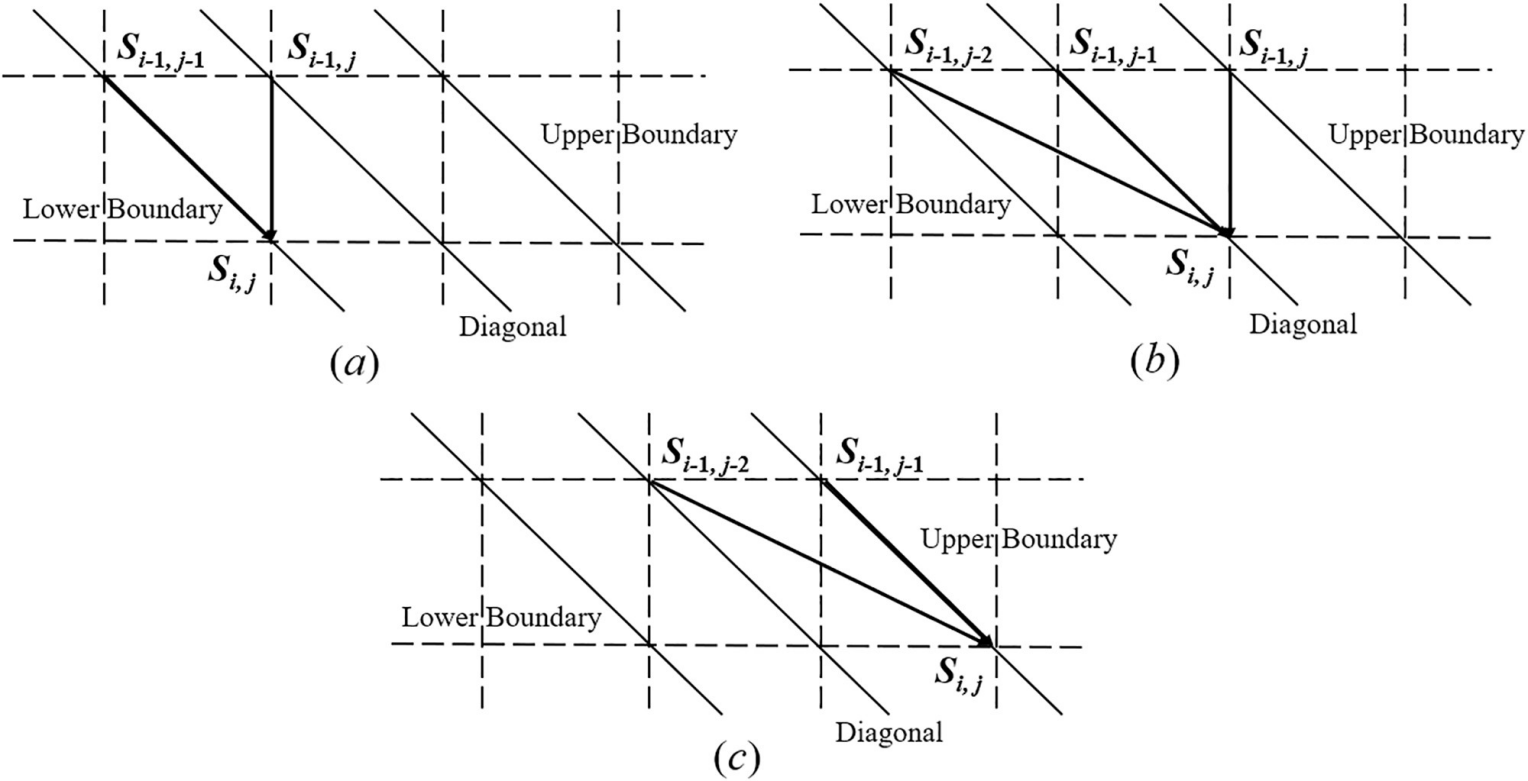
Possible state transitions when *x*_*k*_ is transmitted on condition that *S*_*i*,*j*_ occurs. (a) *S*_*i*,*j*_ is located on the lower boundary (*i* − *j* = 1); (b) *S*_*i*,*j*_ is located on the diagonal (*i* − *j* = 0); (c) *S*_*i*,*j*_ is located on the upper boundary (*j* − *i* = 1).

Suppose *x*_*i*_ is transmitted over the CID channel. According to the relationship between *i* and *j* and the location of the previous state, three scenarios are to be considered in order to calculate *α*_*i*,*j*_ as shown in Fig 5.

**Case 1**: *S*_*i*,*j*_ **is located on the lower boundary when** *i* − *j* = 1 **and it can be reached from** *S*_*i*−1,*j*−1_ **and** *S*_*i*−1,*j*_, **as shown in** Fig 5(a).

$$\begin{array}{c} \alpha_{i,j}=p_{d}\alpha_{i-1,j}+p_{t}^{\prime }\alpha_{i-1,j-1}T\left(x_{i},y_{j}\right). \end{array}$$ (9)

**Case 2**: *S*_*i*,*j*_ **is located on the diagonal when** *i* − *j* = 0 **and it can be reached from** *S*_*i*−1,*j*−2_, *S*_*i*−1,*j*−1_ **and** *S*_*i*−1,*j*_, **as shown in** Fig 5(b).

$$\begin{array}{c} \begin{array}{cc} \alpha_{i,j} & =p_{d}^{\prime }\alpha_{i-1,j}+p_{t}\alpha_{i-1,j-1}T\left(x_{i},y_{j}\right)\;+p_{i}^{\prime }\alpha_{i-1,j-2}T\left(x_{i},y_{j}\right)\text{Pr}\left(\text{inserted}\;\text{bit}=y_{j-1}\right)\\ & =p_{d}^{\prime }\alpha_{i-1,j}+p_{t}\alpha_{i-1,j-1}T\left(x_{i},y_{j}\right)\;+\frac{p_{i}^{\prime }}{2}\alpha_{i-1,j-2}T\left(x_{i},y_{j}\right). \end{array} \end{array}$$ (10)

**Case 3**: *S*_*i*,*j*_ **is located on the upper boundary when** *j* − *i* = 1 **and it can be reached from either** *S*_*i*−1,*j*−2_ **or** *S*_*i*−1,*j*−1_, **as shown in** Fig 5(c).

$$\begin{array}{c} \begin{array}{cc} \alpha_{i,j} & =p_{t}^{\prime }\alpha_{i-1,j-1}T\left(x_{i},y_{j}\right)+p_{i}\alpha_{i-1,j-2}\cdot T\left(x_{i},y_{j}\right)\text{Pr}\left(\text{inserted}\;\text{bit}=y_{j-1}\right)\\ & =p_{t}^{\prime }\alpha_{i-1,j-1}T\left(x_{i},y_{j}\right)+\frac{p_{i}}{2}\alpha_{i-1,j-2}T\left(x_{i},y_{j}\right). \end{array} \end{array}$$ (11)

In summary, given the initial conditions *α*_0,0_ = 1 and *α*_0,1_ = 0, all other values of *α*_*i*,*j*_ within the boundaries in Fig 4 can be calculated iteratively.

### Backward algorithm

Similarly, the backward quantity *β*_*i*,*j*_ denotes the probability that the remaining *r*−*j* received bits are $y_{j+1}^{r}=\left(y_{j+1},\ldots ,y_{r}\right)$ given the transmission state *S*_*i*,*j*_ has occurred. The backward quantity *β*_*i*,*j*_ is therefore denoted by

$$\begin{array}{c} \beta_{i,j}=\text{Pr}\left(y_{j+1}^{r}|S_{i,j}\right). \end{array}$$ (12)

**Fig 6:**
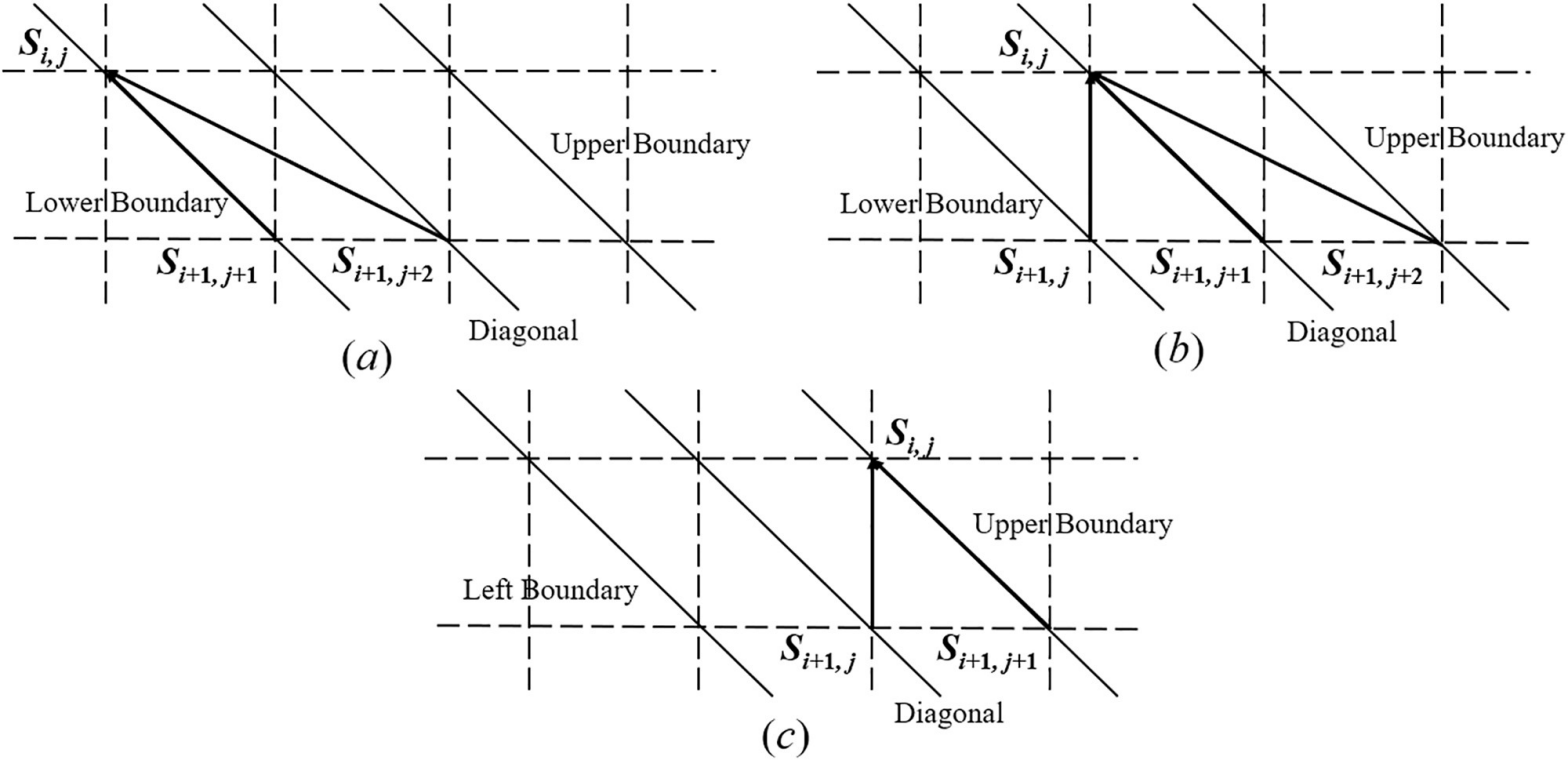
Possible state transitions when *x*_*i*+1_ is transmitted on condition that *S*_*i*,*j*_ has occurred. (a) *S*_*i*,*j*_ is located on the lower boundary (*i* − *j* = 1); (b) *S*_*i*,*j*_ is located on the diagonal (*j* − *i* = 0); (c) *S*_*i*,*j*_ is located on the upper boundary (*j* − *i* = 1).

Suppose *x*_*i*+1_ is transmitted through CID channel. According to the relationship between *i* and *j*, three scenarios are to be considered in order to calculate *β*_*i*,*j*_ as shown in Fig 6. Moreover, the channel error types and accompanying synchronization error probabilities of each transition over the CID channel depends on the location of *S*_*i*,*j*_.

**Case 1**: *S*_*i*,*j*_ **is located on the lower boundary when** *i* − *j* = 1 **and it can transit to** *S*_*i*+1,*j*+1_ **or** *S*_*i*+1,*j*+2_, **as shown in** Fig 6(a).

$$\begin{array}{c} \begin{array}{cc} \beta_{i,j} & =p_{t}^{\prime }\beta_{i+1,j+1}T\left(x_{i+1},y_{j+1}\right)+p_{i}^{\prime }\beta_{i+1,j+2}\cdot T\left(x_{i+1},y_{j+2}\right)\text{Pr}\left(\text{inserted}\;\text{bit}=y_{j+1}\right)\\ & =p_{t}^{\prime }\beta_{i+1,j+1}T\left(x_{i+1},y_{j+1}\right)+\frac{p_{i}^{\prime }}{2}\beta_{i+1,j+2}T\left(x_{i+1},y_{j+2}\right). \end{array} \end{array}$$ (13)

**Case 2**: *S*_*i*,*j*_ **is located on the diagonal when** *i* − *j* = 0 **and it can transit to** *S*_*i*+1,*j*_, *S*_*i*+1,*j*+1_ **or** *S*_*i*+1,*j*+2_, **as shown in** Fig 6(b).

$$\begin{array}{c} \begin{array}{cc} \beta_{i,j} & =p_{d}\beta_{i+1,j}+p_{t}\beta_{i+1,j+1}T\left(x_{i+1},y_{j+1}\right)+p_{i}\beta_{i+1,j+2}T\left(x_{i+1},y_{j+2}\right)\text{Pr}\left(\text{inserted}\;\text{bit}=y_{j+1}\right)\\ & =p_{d}\beta_{i+1,j}+p_{t}\beta_{i+1,j+1}T\left(x_{i+1},y_{j+1}\right)+\frac{p_{i}}{2}\beta_{i+1,j+2}T\left(x_{i+1},y_{j+2}\right). \end{array} \end{array}$$ (14)

**Case 3**: *S*_*i*,*j*_ **is located on the upper boundary when** *j* − *i* = 1 **and it can transit to** *S*_*i*+1,*j*_ **or** *S*_*i*+1,*j*+1_, **as shown in** Fig 6(c).

$$\begin{array}{c} \beta_{i,j}=p_{d}^{\prime }\beta_{i+1,j}+p_{t}^{\prime }\beta_{i+1,j+1}T\left(x_{i+1},y_{j+1}\right). \end{array}$$ (15)

In summary, given the initial conditions *β*_*t*,*r*_ = 1 and *β*_*t*,*r*−1_ = 0, all other values of *β*_*i*,*j*_ within the boundaries in Fig 4 can be calculated iteratively.

### Likelihood

The likelihoods $p(y_{1}^{r}|x_{i})$ are first derived in terms of *α*_*i*,*j*_ and *β*_*i*,*j*_ under three cases, i.e., *x*_*i*_ is deleted; *x*_*i*_ is transmitted without deletion or insertion error; and *x*_*i*_ suffers from an insertion error. They are derived as follows.

**Case 1**: **Only two scenarios are possible when** *x*_*i*_ **is deleted**.

$$p(y_{1}^{r}|x_{i}\;\;\text{is}\;\text{deleted})=\;p_{d}\alpha_{i-1,j}\beta_{i,j}{{|}_{j=i-1}+p_{d}^{\prime }\alpha_{i-1,j}\beta_{i,j}|}_{j=i}.$$ (16)

**Case 2**: **Three scenarios are possible when** *x*_*i*_ **is transmitted without synchronization errors**.

$$\begin{array}{c} \begin{array}{cc} p(y_{1}^{r}|x_{i}\;\; & \text{is}\;\text{transmitted})\\ =\; & p_{t}^{\prime }\alpha_{i-1,j-1}\beta_{i,j}\cdot C\left(x_{i},y_{j}\right){|}_{j=i-1}\\ & +p_{t}\alpha_{i-1,j-1}\beta_{i,j}C\left(x_{i},y_{j}\right){|}_{j=i}\\ & +p_{t}^{\prime }\alpha_{i-1,j-1}\beta_{i,j}C\left(x_{i},y_{j}\right){|}_{j=i+1}. \end{array} \end{array}$$ (17)

**Case 3**: **Only two scenarios are possible when** *x*_*i*_ **is transmitted with an insertion error**.

$$p(y_{1}^{r}|x_{i}\;\;\text{is}\;\text{inserted})=\frac{p_{i}^{\prime }}{2}\alpha_{i-1,j-2}\beta_{i,j}C\left(x_{i},y_{j}\right){{|}_{j=i}+\frac{p_{i}}{2}\alpha_{i-1,j-2}\beta_{i,j}C\left(x_{i},y_{j}\right)|}_{j=i+1}.$$ (18)

Because all the cases are independent, the overall $p(y_{1}^{r}|x_{i})$ is the sum of Eqs (16), (17) and (18), where *x*_*i*_ ∈ {0, 1}. The LLRs are subsequently computed using Eq (5) and are used as soft inputs to the LDPC decoder.

## IV. Results and discussion

Simulations are conducted to investigate the error performance of the concatenated LDPC-marker code over the CID channel. We have implemented the simulation using Matlab program under the Windows operating system and the simulation is carried out as follows. A random sequence of *k* message bits is first generated and encoded into an LDPC codeword of *n* bits. Then markers of length *N*_*m*_ are inserted into an LDPC codeword at a fixed interval *N*_*c*_ to form a block. The encoding procedure is repeated for every *k* message bits as shown in Fig 2. A total of *N* = 10^5^ blocks are generated for simulation. The encoded sequence with *N* blocks is transmitted through the CID channel with insertion probability *p*_*i*_, deletion probability *p*_*d*_, substitution probability *p*_*s*_, constant *A* and common ratio *r*. For all simulations, the (*n* = 4521, *k* = 3552) LDPC code [20] with rate 0.79 is used as the outer code. The code rate of the concatenated code can be determined when the values of *N*_*c*_ and *N*_*m*_ are given.

The received corrupted sequence is first decoded by the inner marker decoder. The inner marker decoder uses the modified forward-backward algorithm described in Sect. IV to derive the LLR of each LDPC code bit. All LLRs are sent to the outer LDPC decoder. The LDPC decoder further decodes the codeword through the sum-product algorithm. At the end of each iteration, the LDPC decoder calculates the updated *a posteriori* LLR for each transmitted bit and make a hard decision on the LLRs to estimate a decoded codeword. The LDPC decoder immediately checks whether the estimated codeword satisfies all check equations. The block is decoded successfully if it satisfies all check equations; otherwise, the iteration continues. In our simulations, the maximum number of iterations is set to 60. If the decoded codeword cannot satisfy all check equations after the maximum number of iterations, the decoding fails. We perform the decoding procedure and count the number of decoded bits and blocks in error. The bit error rate (BER) and block error rate (BLER) are used as metrics to evaluate the error performance of the concatenated LDPC-marker code.

### BER/BLER error performance

**Fig 7:**
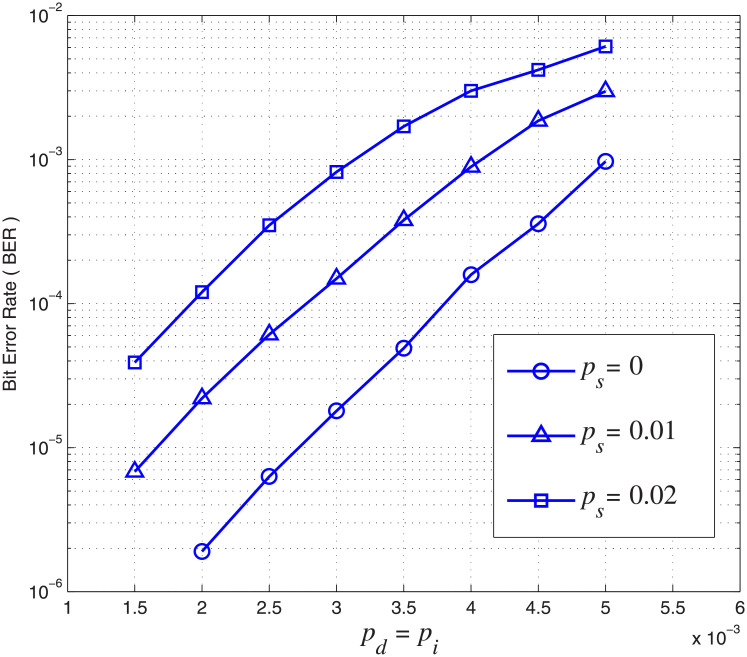
BER of the concatenated LDPC-marker code over the CID channel. *N*_*c*_ = 18 and *N*_*m*_ = 2.

**Fig 8:**
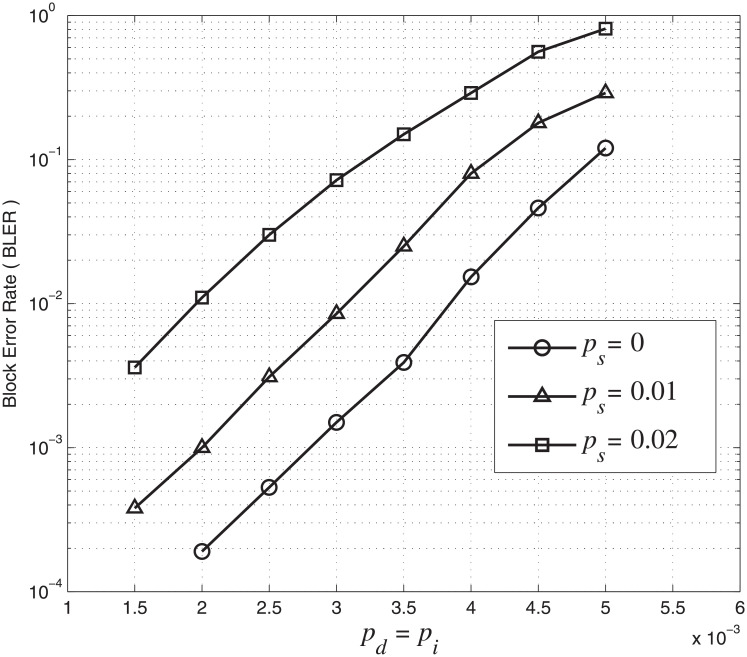
BLER of the concatenated LDPC-marker code over the CID channel. *N*_*c*_ = 18 and *N*_*m*_ = 2.

In the first set of simulations, regular markers ‘10’ of length *N*_*m*_ = 2 are inserted at the beginning of each LDPC codeword, and also periodically every *N*_*c*_ = 18 LDPC code bits. In other words, each marker is followed by 18 LDPC code bits, except the last marker which is followed only by 3 code bits. The total length of the transmitted sequence is 5023 bits per block and the overall code rate is *R* = 3552/5023 = 0.71. For this simulation, we consider *p*_*i*_ = *p*_*d*_ and set *A* = *r* = 0.5 in Eq (1). The BER and BLER performance of the concatenated code over the CID channel are shown in Figs 7 and 8, respectively, under different channel insertion, deletion and substitution error rates. As expected, both BER and BLER increase as *p*_*i*_, *p*_*d*_ and *p*_*s*_ increase. We first investigate the case when there are only insertion and deletion errors, i.e., *p*_*s*_ = 0. The BER of the concatenated LDPC-marker code can be as low as 2 × 10^−6^ when *p*_*i*_ = *p*_*d*_ = 2 × 10^−3^. The BER of the concatenated LDPC-marker code increases as *p*_*i*_ and *p*_*d*_ increase. For example, the BER of the concatenated LDPC-marker code increases from 2 × 10^−6^ to 10^−3^ when *p*_*i*_ and *p*_*d*_ increase from 2 × 10^−3^ to 5 × 10^−3^ in Fig 7. In Fig 8, the BLER of the concatenated LDPC-marker code can be as low as 2 × 10^−4^ when *p*_*i*_ = *p*_*d*_ = 2 × 10^−3^. The BLER of the concatenated LDPC-marker code also increases as *p*_*i*_ and *p*_*d*_ increase. For example, the BLER of the concatenated LDPC-marker code increases from 2 × 10^−4^ to 0.12 when *p*_*i*_ and *p*_*d*_ increase from 2 × 10^−3^ to 5 × 10^−3^ as shown in Fig 8. The result shows that the inner decoder can effectively maintain synchronization over the CID channel with the help of the modified forward-backward algorithm.

Furthermore, we investigate and compare the error performance in terms of BER and BLER with different *p*_*s*_. The simulation results show that the decoder can correctly decode the corrupted block when *p*_*s*_ is relatively low. For example, when *p*_*i*_ = *p*_*d*_ = 3 × 10^−3^ and *p*_*s*_ = 0.01, BER and BLER are as low as 1.7 × 10^−4^ and 8 × 10^−3^, respectively. However, more bits suffer from substitution errors as *p*_*s*_ increases and hence both BER and BLER increases. For example, the BLER increases rapidly from 2 × 10^−4^ to 1.2 × 10^−2^ when *p*_*s*_ increases from 0 to 0.02 (*p*_*i*_ = *p*_*d*_ = 2 × 10^−3^). The corrupted block cannot be decoded at a higher substitution error rate because the number of substitution errors exceeds the correction capability of the LDPC code.

### Impact of markers on error performance

**Fig 9:**
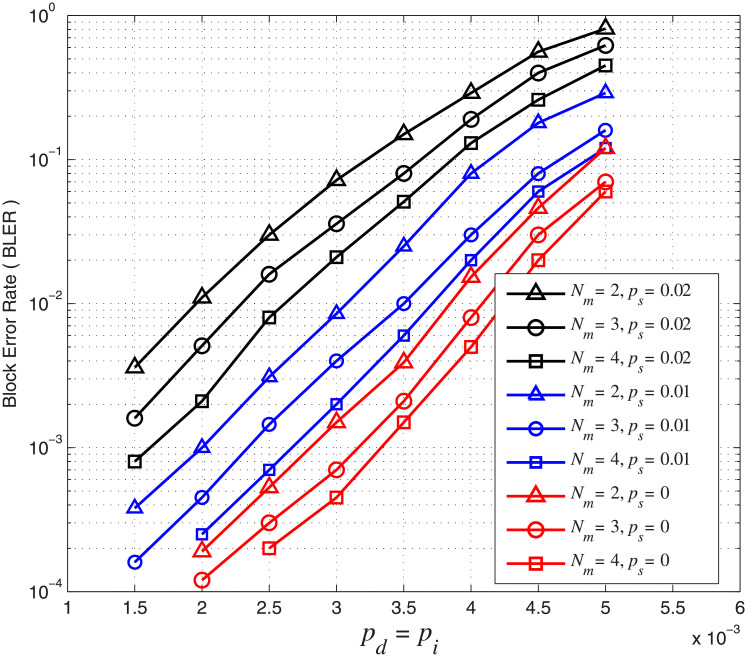
BLER of the concatenated LDPC-marker code over the CID channel. *N*_*c*_ = 18 and *N*_*m*_ = 2, 3, 4.

We first investigate the marker length *N*_*m*_ on the error performance of the concatenated code. We fix *N*_*c*_ = 18 and use *N*_*m*_ = 2, 3, 4. The markers used are ‘10’, ‘101’ and ‘1010’ while the corresponding overall code rates are 0.71, 0.67 and 0.64, respectively. For this simulation, we set *A* = *r* = 0.5 in Eq (1). The BLER performance of concatenated codes with different marker length *N*_*m*_ is shown in Fig 9. The results show that when *N*_*c*_ is fixed, the BLER decreases as the marker length *N*_*m*_ increases. For example, when *p*_*d*_ = *p*_*i*_ = 4 × 10^−3^ and *p*_*s*_ = 0.01, the BLER decreases from 8 × 10^−2^ (*N*_*m*_ = 2) to 2 × 10^−2^ (*N*_*m*_ = 4). When *p*_*d*_ = *p*_*i*_ = 3 × 10^−3^ and *p*_*s*_ = 0, the BLER decreases from 7 × 10^−4^ (*N*_*m*_ = 3) to 4.5 × 10^−4^ (*N*_*m*_ = 4). In other words, the error performance of the concatenated code is improved by increasing the marker length *N*_*m*_. However, the code rate of the concatenated code is decreased as the marker length *N*_*m*_ increases. We also notice that the error performance of the concatenated code in terms of BLER is seriously affected by *p*_*s*_. For example, when *p*_*i*_ = *p*_*d*_ = 3 × 10^−3^, the BLER for *N*_*m*_ = 4 increases by about 50 times, rapidly from 4.5 × 10^−4^ (*p*_*s*_ = 0) to 2.1 × 10^−2^ (*p*_*s*_ = 0.02). The results show that even though markers help to maintain synchronization, corrupted blocks cannot be corrected due to a large number of substitution errors.

**Fig 10:**
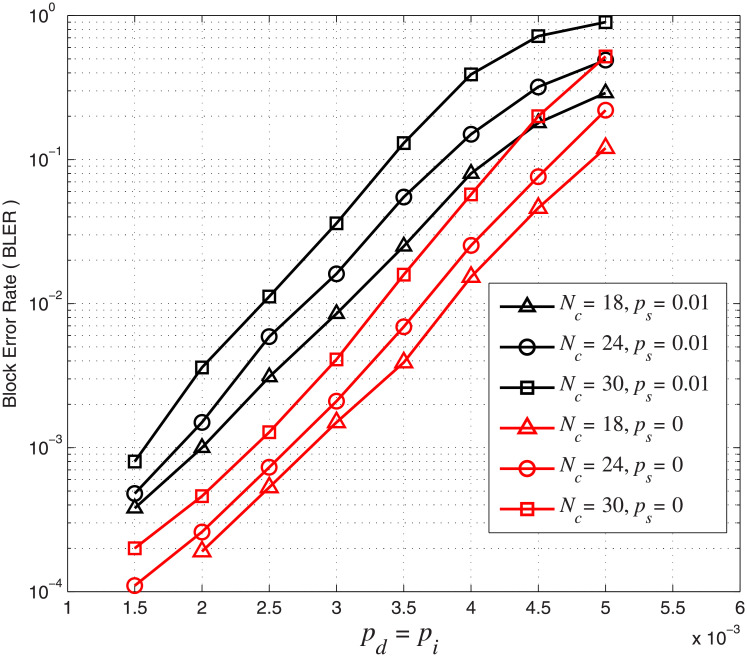
BLER of the concatenated LDPC-marker code over the CID channel. *N*_*m*_ = 2 and *N*_*c*_ = 18, 24, 30.

We also study the effect of marker interval *N*_*c*_ on the error performance of the concatenated code. We fix *N*_*m*_ = 2 and use *N*_*c*_ = 18, 24, 30. The corresponding overall code rates are 0.71, 0.73 and 0.74, respectively. The marker interval *N*_*c*_ is related to the number of markers. A larger marker interval means fewer inserted markers in a block. For this simulation, we set *A* = *r* = 0.5 in Eq (1). In Fig 10, we plot BLER curves for concatenated codes with different marker intervals *N*_*c*_ under different channel parameters. It can be observed that the BLER error performance is improved with a smaller marker interval or more markers. For example, when *p*_*d*_ = *p*_*i*_ = 3 × 10^−3^ and *p*_*s*_ = 0, the BLER increases from 2.1 × 10^−3^ (*N*_*c*_ = 24) to 4 × 10^−3^ (*N*_*c*_ = 30). When *p*_*d*_ = *p*_*i*_ = 2.5 × 10^−3^ and *p*_*s*_ = 0.01, the BLER increases from 3 × 10^−3^ (*N*_*c*_ = 18) to 5.8 × 10^−3^ (*N*_*c*_ = 24). In other words, reducing the marker interval or inserting more markers in a block improves the error performance of the concatenated LDPC-marker code.

From the simulation results in Figs 9 and 10, we conclude that codes with larger marker length *N*_*m*_, smaller marker interval *N*_*c*_ and more markers have a better synchronization capability and hence error performance. Although increasing *N*_*m*_ and/or reducing *N*_*c*_ improve the error performance of the concatenated code, the information transmission efficiency and code rate is reduced.

### Comparison with existing schemes

**Fig 11:**
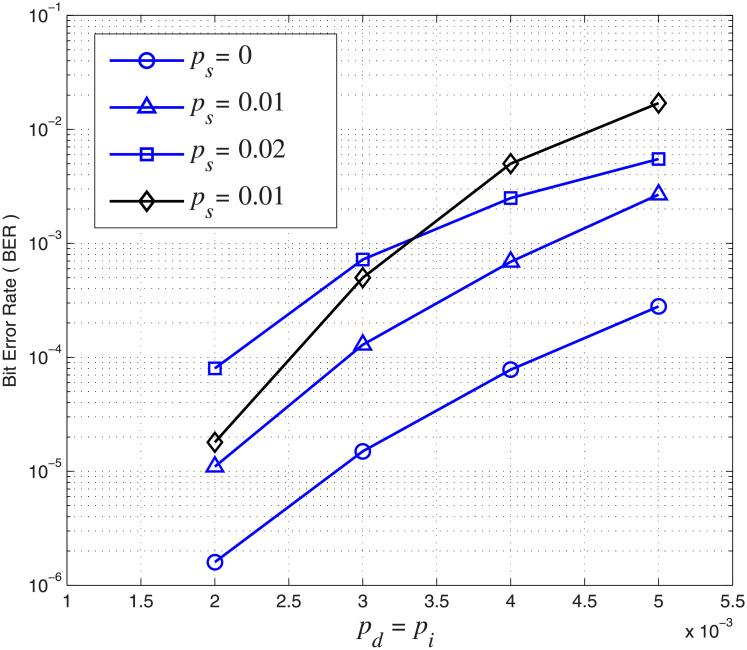
Comparision performance under different channel parameters. *p*_*d*_ = *p*_*i*_ increase from 2 × 10^−3^ to 5 × 10^−3^ and *p*_*s*_ increases from 0 to 0.02. Blue curves represent concatenated LDPC-marker codes under different *p*_*s*_ and the black curve represents the best code in [16] when *p*_*s*_ = 0.01.

Simulations are further carried out to compare the error performance of the concatenated LDPC-marker code with the result in [16]. In order to make a fair comparison, we set *A* = 0.5 and *r* = 1 in Eq (1). Thus, $p_{d}^{\prime }=p_{i}^{\prime }=0.5$ are consistent with the error rates in [16] for subsequent bits after a synchronization error. Furthermore, the inner marker code with *N*_*m*_ = 2 and *N*_*c*_ = 18 is adopted so that both codes have the same code rate 0.71 for a fair comparison. The BLER curves of the concatenated LDPC-marker code over the CID channel and the BLER curve with the best performance in [16] under the same channel parameters are compared in Fig 11. The simulation result shows that the concatenated LDPC-marker code performs much better than the best code in [16] when *p*_*s*_ = 0.01. This improvement is more significant with the increase of insertion and deletion error rates. For example, when *p*_*d*_ = *p*_*i*_ = 5 × 10^−3^ and *p*_*s*_ = 0.01, the BER of the concatenated code is 2.8 × 10^−3^ while the BER of the best code in [16] is more than 1 × 10^−2^. We also observe that the concatenated code at *p*_*s*_ = 0.02 even performs better than the best code in [16] at *p*_*s*_ = 0.01 when *p*_*d*_ and *p*_*i*_ are greater than 3.3 × 10^−3^. This proves that the concatenated LDPC-marker code has a better resistance to substitution errors. Compared with [16], the CID channel model is more accurate for BPMR systems and the error performance is improved under the same channel parameters.

**Fig 12:**
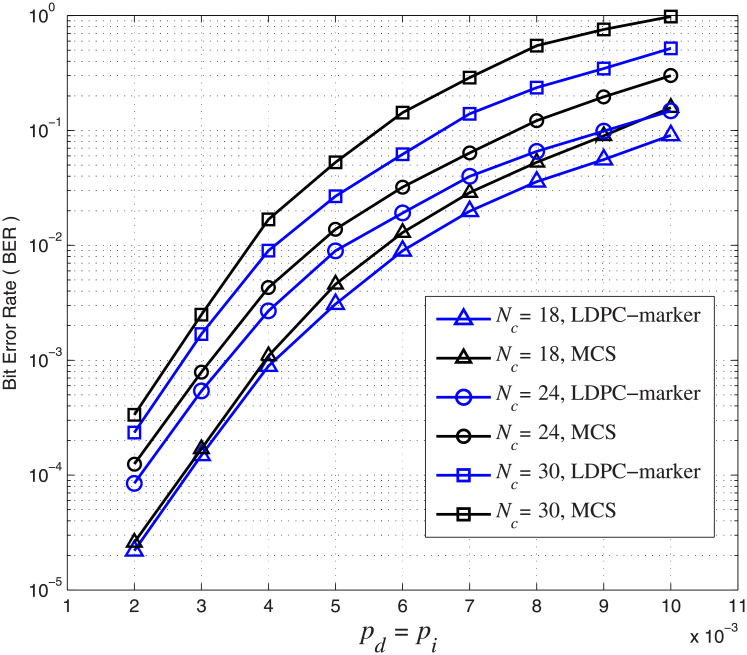
BER performance comparison over the CID channel. *p*_*d*_ = *p*_*i*_ increase from 2 × 10^−3^ to 10^−2^ and *p*_*s*_ = 0.01. The blue curve represents the concatenated LDPC-marker code and the black curve represents the conventional marker coding scheme.

**Fig 13:**
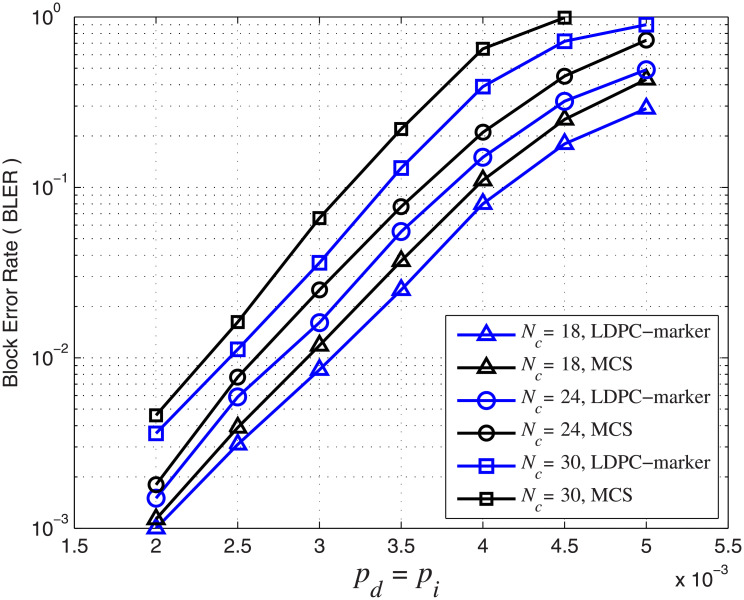
BLER performance comparison over the CID channel. *p*_*d*_ = *p*_*i*_ increase from 2 × 10^−3^ to 5 × 10^−3^ and *p*_*s*_ = 0.01. The blue curve represents the concatenated LDPC-marker code and the black curve represents the conventional marker coding scheme.

We further investigate and compare the error performance of the concatenated LDPC-marker code considering correlated synchronization errors with the conventional marker coding scheme (MCS) [8] without considering the dependency between synchronization errors over the CID channel. In the following, we use the (4521, 3552) LDPC code as the outer code. For the inner marker code, we fix *N*_*m*_ = 2 and use *N*_*c*_ = 18, 24, 30. The corresponding overall code rates are 0.71, 0.73 and 0.74, respectively. For this simulation, we consider *p*_*i*_ = *p*_*d*_, *p*_*s*_ = 0.01 and set *A* = *r* = 0.5 in Eq (1). We simulate the error performance of the two coding schemes. The BER and BLER error performance over the CID channel are shown in Figs 12 and 13, respectively.

The simulation result shows that the concatenated LDPC-marker code produces a lower BER/BLER error rate than the conventional MCS over the CID channel. The improvement is intuitive and can be explained as follows. The conventional MCS assumes that insertion, deletion and substitution errors occur randomly without correlation, and it does not consider the dependency between the synchronization errors in the CID channel. On the contrary, the concatenated LDPC-marker code takes into account the correlation to compute LLRs by means of the modified forward-backward algorithm. As a result, LLRs provided by the inner decoder of the concatenated LDPC-marker code are more accurate. In addition to error performance, we compare the decoding complexity of the two coding schemes. Compared with the MCS, the decoding time of the concatenated LDPC-marker code is greatly reduced by 35% on average for each BLER curve in Fig 13. The explanation is as follows. The width of the decoding trellis is proportional to insertion and deletion error rates. As insertion and deletion error rates increase, the scale of the decoding trellis becomes larger and the computational complexity becomes higher. For the concatenated LDPC-marker code, the decoding trellis is limited to a small range (between upper boundary and lower boundary) because insertion and deletion errors always occur in pairs. Many transitions on the transition diagram are not allowed due to the correlation between synchronization errors. For example, consecutive insertions/deletions are not allowed in the CID channel. However, the decoding scale of the MCS is relatively large and the decoder has to find the mostly likely transmission path among all possible candidates.

## V. Conclusion and future work

In this paper, we have proposed a probabilistic channel model suitable for BPMR systems with frequent correlated insertion/deletion errors. We have further modified the forward-backward algorithm and investigated the error performance of a concatenated code over this channel. The simulation results show that the inner marker code can effectively maintain synchronization, while the outer LDPC code can provide sufficient error correction capability for the BPMR system. The error performance can be further improved when the marker interval is reduced and/or more markers are inserted in the transmitted sequence. The simulation result shows that the concatenated LDPC-marker code performs much better than the code in [16]. The improvement is more significant with the increase of insertion and deletion error rates. We also investigate and compare the error performance of the concatenated LDPC-marker code considering correlated synchronization errors with the existing conventional MCS without considering the dependency between synchronization errors over the CID channel. The simulation result shows that the concatenated LDPC-marker code produces a lower BER/BLER error rate than the conventional MCS over the CID channel. Compared with existing methods, our coding scheme improves the error performance and reduces the decoding complexity. We have assumed binary data transmission and the fixed LDPC code in this study. In the future, we plan to investigate *M*-ary data transmission and different LDPC codes over the CID channel.
